# Rapidly repeated visual stimulation induces long-term potentiation of VEPs and increased content of membrane AMPA and NMDA receptors in the V1 cortex of cats

**DOI:** 10.3389/fnins.2024.1386801

**Published:** 2024-05-20

**Authors:** Shunshun Chen, Hongyan Lu, Changning Cheng, Zheng Ye, Tianmiao Hua

**Affiliations:** College of Life Sciences, Anhui Normal University, Wuhu, Anhui, China

**Keywords:** repeated high-frequency visual stimulation, long-term potentiation, visually-evoked field potentials, AMPA and NMDA receptors, primary visual cortex, cat

## Abstract

Studies report that rapidly repeated sensory stimulation can evoke LTP-like improvement of neural response in the sensory cortex. Whether this neural response potentiation is similar to the classic LTP induced by presynaptic electrical stimulation remains unclear. This study examined the effects of repeated high-frequency (9 Hz) versus low-frequency (1 Hz) visual stimulation on visually-evoked field potentials (VEPs) and the membrane protein content of AMPA / NMDA receptors in the primary visual cortex (V1) of cats. The results showed that repeated high-frequency visual stimulation (HFS) caused a long-term improvement in peak-to-peak amplitude of V1-cortical VEPs in response to visual stimuli at HFS-stimulated orientation (SO: 90°) and non-stimulated orientation (NSO: 180°), but the effect exhibited variations depending on stimulus orientation: the amplitude increase of VEPs in response to visual stimuli at SO was larger, reached a maximum earlier and lasted longer than at NSO. By contrast, repeated low-frequency visual stimulation (LFS) had not significantly affected the amplitude of V1-cortical VEPs in response to visual stimuli at both SO and NSO. Furthermore, the membrane protein content of the key subunit GluA1 of AMPA receptors and main subunit NR1 of AMPA receptors in V1 cortex was significantly increased after HFS but not LFS when compared with that of control cats. Taken together, these results indicate that HFS can induce LTP-like improvement of VEPs and an increase in membrane protein of AMPA and NMDA receptors in the V1 cortex of cats, which is similar to but less specific to stimulus orientation than the classic LTP.

## 1 Introduction

A long-lasting increase in the amplitude of postsynaptic potentials after rapidly repeated electrical stimulation at the presynaptic fiber is known as the long-term potentiation (LTP) of synaptic transmission for about a half century (Bliss and Lomo, 1973; Douglas and Goddard, 1975; Sarvey et al., 1989; Colbert and Levy, 1993; Malenka and Nicoll, 1999; Matsuzaki et al., 2004). For the potentiation of postsynaptic potentials shows a high input-specificity and is induced only at synapses stimulated by afferent activity (Kelso et al., 1986; Colbert and Levy, 1993; Kirkwood and Bear, 1994; Volianskis and Jensen, 2003), LTP is widely regarded as a form of synaptic plasticity that mediates learning and memory (Bliss and Collingridge, 1993; Malenka and Nicoll, 1999; Muller et al., 2002; Liu et al., 2004; Cooke and Bliss, 2006; Hager and Dringenberg, 2010; Sale et al., 2011; Aberg and Herzog, 2012; Sumner et al., 2020a). Therefore, since its discovery in the hippocampus (Bliss and Lomo, 1973; Kelso et al., 1986), LTP has attracted a considerable attention in neuroscience researches and has been found to occur widely in the brain regions, including sensory cortex (Berry et al., 1989; Bear et al., 1992; Kirkwood and Bear, 1994; Kudoh and Shibuki, 1994; Sale et al., 2011) and associative cortex (Jay et al., 1995; Chen et al., 1996).

The cellular and molecular mechanism underlying LTP has been extensively studied in the past decades (Bliss et al., 1986; Sarvey et al., 1989; Bliss and Collingridge, 1993; Kirkwood and Bear, 1994; Jay et al., 1995; Kessey and Mogul, 1997; Malenka and Nicoll, 1999; Lynch et al., 2000; Liu et al., 2004; Matsuzaki et al., 2004). It is known that high-frequency repeated presynaptic stimulation can increase the release of glutamate, which improves depolarization across the postsynaptic membrane through activation of more AMPA receptors and thus allows opening of NMDA channels by Mg^2+^ displacement from NMDA receptors. Influx of Ca^2+^ ions through NMDA channels triggers cascades of second-messenger systems within the postsynaptic neuron and thus causes the LTP by increasing the assembling of AMPA receptors at postsynaptic membrane (Miyamoto, 2006; Zhong et al., 2006; Peng et al., 2010).

Although LTP is a well-known synaptic plasticity, most of its knowledge comes from animal studies using invasive electrical presynaptic stimulation (Bliss and Lomo, 1973; Teyler and DiScenna, 1987; Kirkwood and Bear, 1994; Jay et al., 1995; Murphy et al., 1997; Malenka and Nicoll, 1999; Matsuzaki et al., 2004; Miyamoto, 2006; Müller et al., 2009; Peng et al., 2010; France et al., 2022). Researches in recent years report that repeated presentation of noninvasive high-frequency visual and auditory stimuli can also induce LTP-like long-lasting increase of neural activity in the visual and auditory cortex of humans and rodents (Teyler et al., 2005; Cooke and Bliss, 2006; McNair et al., 2006; Ross et al., 2008; Zheng et al., 2008; Clapp et al., 2012; Sanders et al., 2018; Sumner et al., 2020a; Dias et al., 2022). However, whether this LTP-like neural response plasticity displays a property identical to the classic LTP induced by presynaptic electrical stimulation is not fully conformed (Rygvold et al., 2021; Dias et al., 2022), and its cellular and molecular mechanism remains considerably unclear (Sanders et al., 2018; Sumner et al., 2020b; Rygvold et al., 2021; Dias et al., 2022). A few of investigations in rodents show that repeated high-frequency visual stimulation can potentiate the visually evoked field potentials (VEPs) (Cooke and Bear, 2010) or increase the expression of extrasynaptic glutamatergic receptors (Eckert et al., 2013), and sensory-evoked neural response potentiation can be blocked by antagonists of NMDA and AMPA receptors (Clapp et al., 2006, 2012). These reports suggest that visual stimulation induced LTP-like effect may share a similar mechanism with the classic LTP. However, direct evidence is lacking so far.

To clarify the issues above, this study attempts to examine the VEPs (Teyler et al., 2005; Ross et al., 2008) in the primary visual cortex (V1) of cats before and after repeated high-frequency visual stimulation (HFS) and low-frequency visual stimulation (LFS), respectively, so as to explore the property of visual stimulation-induced LTP-like neural response changes. Concurrently, we will measure the alterations in membrane protein content of AMPA and NMDA receptors in the V1 cortex after HFS or LFS, trying to see if the visual stimulation-induced LTP-like neural response improvement is mediated by a mechanism similar to the classic LTP.

## 2 Materials and methods

### 2.1 Subjects

A total of 26 cats (aged 1–3 years and weighing 2.5–3.2 kg) were used as subjects in this study. All cats were purchased from Nanjing Qing-Long-Shan Animal Breeding Farm (Jiangning District of Nanjing, Certificate No. SX1207) and all of them were disease-free, healthy subjects with no optical or retinal abnormality. All animals were reared in rooms separated by transparent glass walls. Each room had comfortably organized living, feeding, and playing areas, and the room temperature was maintained at 25°C. The cats could get clean food and water freely. Each animal was fasted for 12 h before the experiment. Eight cats were randomly selected for electrophysiological experiments to examine, respectively, the effects of HFS (4 cats) and LFS (4 cats) on visually-evoked field potentials (VEPs) in the primary visual cortex (V1: area 17), and 18 cats were used for Western blot experiments to assess the membrane protein content of glutamatergic AMPA and NMDA receptors in the V1 cortex after HFS (6 cats) or LFS (6 cats) versus that of control cats (6 cats).

All experiments in this study were performed strictly in accordance with the National Institutes of Health Guide for the Care and Use of Laboratory Animals, and conformed to the principles and regulations as described in the ARRIVE guidelines (Animal Research: Reporting of *In Vivo* Experiments). All experiments and animal treatments were approved by the Ethics Committee of Anhui Normal University (approval No: AHNU-ET 2023015).

### 2.2 VEPs recording in the V1 cortex before and after HFS or LFS

#### 2.2.1 Recording preparation

The preparation for recording of VEPs in the V1 cortex was performed with the following procedures according to previous studies (Hua et al., 2010; Zhao et al., 2020; Ding et al., 2022; Yu et al., 2023). The cat was first anesthetized with ketamine HCl (40 mg/kg, im) and xylazine (2 mg/kg, im). Noninvasive intubation of tracheal and intravenous cannula was performed under sterile preparation. After the cat was fixed in a stereotaxic apparatus, glucose (5%)-saline (0.9%) solution containing a mixture of urethane (20 mg/kg body weight) and gallamine triethiodide (10 mg/kg body weight) was infused intravenously to maintain necessary anesthesia and paralysis. Artificial respiration was performed, and the expired pCO_2_ was kept at approximately 3.8%. The animal’s electrocardiogram, heart rate (180–220 beats/min), and blood oxygen level (>95%) were monitored continuously throughout the experiment to evaluate the anesthesia level and physiological state. The body temperature (38°C) was maintained using a heating blanket. Pupils were maximally dilated with atropine (0.5%). Artificial tear was applied to protect the cornea from dryness.

A small hole (4 × 3 mm) was drilled on the skull over the central V1 area (Horsley–Clarke coordinates: P2-6/L2-4) of the left hemisphere. A glass-coated silver wire electrode (extending from P2 to P6, with an impedance of 0.3–0.5 MΩ) was implanted on the surface of the dura over V1 area for VEP recording. The exposed small hole was filled with 4% agar, sealed with tissue adhesive and fixed with dental cement.

#### 2.2.2 VEP recording procedures and visual stimuli

VEP signals in V1 cortex before and after repeated presentation (2,700 trials) of high-frequency (9 Hz) or low-frequency (1 Hz) flickering grating stimuli (full screen size, with orientation 90°, spatial frequency 0.2 cpd and contrast 100%) were recorded using the embedded silver wire electrode. Signals were amplified with a microelectrode amplifier (Dagan 2400A, Minneapolis, MN, USA) (gain 1,000, band-pass filtered between 1 and 200 Hz), digitized with an acquisition board (National Instruments, USA) controlled by IGOR software (WaveMetrics, USA) and then saved for on- or off- line analysis.

To avoid any overlap of effects from HFS and LFS, each cat received only HFS or LFS. The experiments for HFS or LFS in each cat repeated 6 times with an interval of at least 4 h. During each experiment, changes of VEPs in V1 cortex before and at different time point (0, 15, 30, 45, 60, 75, 90, 105, 120, 135, 150, 165, 180 min) after the end of HFS or LFS were assessed using test visual stimuli of flickering gratings (full screen size, with vertical or horizontal oriented orientation, spatial frequency 0.2 cpd, temporal frequency 0.5 Hz and contrast 100%). The vertical and horizontal oriented grating stimuli were presented in an interleaved order and repeated 3 iterations, with 6 trials per iteration. The duration of each stimulus presentation was 0.5 s, and the baseline of local field potential were acquired during 1 s pre-stimulus interval in which the still grating image was shown on the CRT.

Visual stimuli were generated by a PC computer using Matlab programs (MathWorks Inc., Natick, MA, USA) based on Psychotoolbox extensions (psychtoolbox.org) (Brainard, 1997), and were presented on a CRT (resolution 1,024 × 768 pixels, refresh rate 75 Hz) positioned 57 cm from the animal’s eyes. VEP signals recoded before and at different time point (0–180 min) after the end of repeated HFS or LFS were averaged across 18 trials and filtered (60 Hz notch filter, 1–200 Hz bandpass) using IGOR programs, and the peak-to-peak amplitude N1P1 and P1N2 of VEPs were measured, respectively.

At the end of VEPs recording, the animals were euthanized by stopping its heart beat and breath through intravenous injection of pentobarbital sodium (>100 mg/kg).

### 2.3 Measurement of protein content with Western blot assays

Brain tissues containing V1 cortex (area 17) were collected 30 min after the end of repeated HFS or LFS. The membrane protein content of the key subunit GluA1 of AMPA receptors and the main subunit NR1 of NMDA receptors in the V1 cortex after repeated HFS or LFS versus controls was measured using Western blot techniques.

Western blot assays were conducted using methods similar to those in our previous studies (Yang et al., 2016; Ding et al., 2017; Zhao et al., 2020; Zhang et al., 2022). The frozen tissues of V1 cortex were cut, weighed, thawed, homogenized in 10 volumes of an ice-cold buffer [25-mM Tris–HCl (pH: 7.6), 150 mM NaCl, 1% NP-40, 1% sodium deoxycholate, and 0.1% SDS] and a protease inhibitor cocktail (Beyotime Biotechnology, Shanghai, China), and spun down at 12,000 rpm for 15 min at 4°C. The supernatant was saved and its protein concentration was assessed using BCA protein quantification kit (Beyotime Biotechnology, Shanghai, China, #P0010S). The plasma membrane proteins (the expression of glutamatergic receptors’ subunits in the plasma membrane) were prepared using a Membrane Protein Extraction Kit (Beyotime Biotechnology, Shanghai, China, #P0033) containing protease inhibitor cocktail and phosphatase inhibitor cocktail, and homogenized with 20 full strokes in glass homogenizers. The lysates were centrifuged for 700 g at 4°C for 10 min. The supernatant was centrifuged again at 14,000 g at 4°C for 30 min. The pellet was resuspended in the lysis buffer and was centrifuged for 14,000 g at 4°C for 5 min. The protein concentration was assessed using BCA protein quantification kit (Beyotime Biotechnology, Shanghai, China, #P0010S). We fractionated the proteins (30 μg) from each sample using 8% or 10% sodium dodecyl sulfate polyacrylamide gel electrophoresis and transferred them onto polyvinylidene fluoride membranes (Beyotime Biotechnology). The membranes were blocked with 5% non-fat dry milk in TBS-Tween 20 for 1 h and incubated overnight at 4°C in TBS-Tween 20 containing primary antibodies, including rabbit anti-GluA1 (1: 1000, Cell Signaling Technology, #13185), rabbit anti-NR1 (1: 1,000, Abcam, #ab17345), and rabbit anti-β-Tubulin (1:2000, Affinity Biosciences, #AF7011). They were then washed three times for 10 min in TBS-Tween 20, incubated with peroxidase conjugated affinipure goat anti-rabbit IgG (1:8,000, Sangon Biotechnology, China, D10058) diluted in TBS-Tween 20 for 2 h at 25°C, and washed again in TBS-Tween 20.

Images of Western blot bands were captured by Tanon 5,200 Multi chemiluminescent imaging system (Tanon, Shanghai, China) (Li et al., 2019; Xu et al., 2020; Zhang et al., 2022). The optical density (OD) of western blot bands was measured using Image J software (National Institutes of Health, Montgomery, Bethesda, MA, USA). The OD value of GluA1 and NR1 bands were normalized against the OD values of the corresponding β- Tubulin bands in each sample, respectively.

### 2.4 Statistical analysis

All value were shown as individual measurement value and mean ± SD. Statistical comparison of VEPs amplitude before and after repeated HFS or LFS as well as the normalized OD of GluA1 and NR1 in the V1 cortex between groups was done using ANOVA and LSD (least significance difference) *Post hoc* pairwise tests (Zhao et al., 2020; Ding et al., 2022).

## 3 Results

### 3.1 Effects of repeated HFS and LFS on VEPs in the V1 cortex

**Figure 1:**
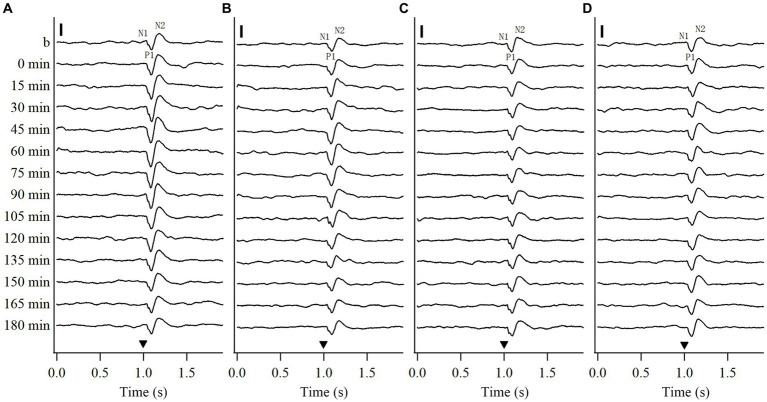
Voltage trace samples showing VEPs of V1 cortex in response to test visual stimuli with stimulated orientation (SO: 90°) and non-stimulated orientation (NSO: 180°) before and at different time point after the end of repeated HFS **(A, B)** or LFS **(C, D)**. The horizontal axis in **(A–D)** shows the recording time (s): the filled triangle denotes the onset of test visual stimuli with orientation at SO **(A,C)** or NSO **(B,D)**, spatial frequency 0.2 cpd, temporal frequency 0.5 Hz and contrast 100%. The baseline field potential is acquired during 1 s before onset of test visual stimuli. The vertical axis displays the recording time points, including before and at 0, 15, 30, 45, 60, 75, 90, 105, 120, 135, 150, 165, and 180 min after the end of HFS or LFS. The VEP contains three main components of wave N1, P1 and N2. The vertical scale bar represents 100 μv.

To explore whether repeated HFS and LFS can induce a long-term potentiation of VEPs in the V1 cortex, this study recorded the VEPs of V1 cortex in response to test visual stimuli (see Methods 2.2.2) before and at different time point (0, 15, 30, 45, 60, 75, 90, 105, 120, 135, 150, 165, 180 min) after the end of repeated HFS or LFS. To examine if the effects induced by repeated HFS and LFS were specific to the stimulated orientation, we recorded the VEPs of V1 cortex in response to test visual stimuli at the stimulated orientation (SO: 90°) and at non-stimulated orientation (NSO: 180°) orthogonal to the SO both before and after HFS or LFS. The voltage-traces of VEPs in response to test visual stimuli at SO and NSO recorded both before and after repeated HFS and LFS were similar in components, containing wave N1, P1 and N2 (Figure 1). The latencies in the peak wave N1, P1 and N2 of VEPs showed no evident alteration after repeated HFS and LFS (Figures 1A–D). However, the amplitude of VEPs was increased after the end of HFS at first, and then returned to the level before HFS (Figures 1A,B). The amplitude of VEPs had no evident change before and after repeated LFS (Figures 1C,D).

#### 3.1.1 Effects of repeated HFS on VEPs in V1 cortex

**Figure 2:**
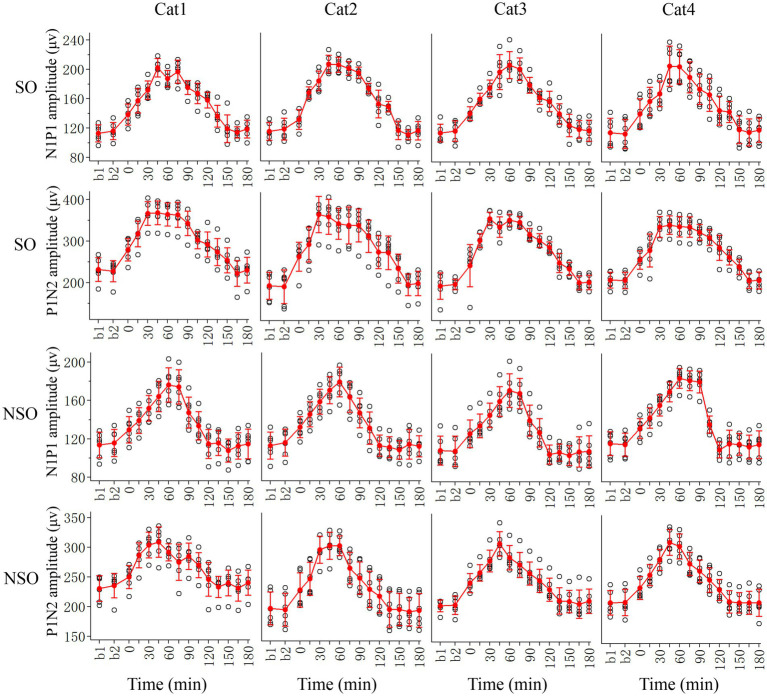
Showing alterations in peak-to-peak amplitude N1P1 and P1N2 of VEPs at V1 cortex of 4 cats (Cat1-4) in response to test visual stimuli with stimulated orientation (SO: 90°) and non-stimulated orientation (NSO: 180°) before (b1, b2) and at different time point (0–180 min, with an interval of 15 min) after the end of repeated HFS. The red filled circle with an error bar represents the mean VEP amplitude of N1P1 and P1N2 with standard deviation (SD), and the open black circles represent individual data of N1P1 and P1N2 value from 6 repeated experiments. Each individual data of VEP amplitude is measured across 18 trials (3 iteration with 6 trials per iteration) of test visual stimuli before and at different time point after HFS.

We first examined the effects of repeated HFS on the peak-to-peak amplitude N1P1 and P1N2 of VEPs of V1 cortex in response to test visual stimuli with orientation at SO (90°) and NSO (180°) orthogonal to the SO (Figure 2).

Before HFS, the amplitude of VEPs of V1 cortex in response to visual stimuli with SO had no significant difference from that with NSO [N1P1: *F*(1, 96) = 0.282, *p* = 0.597; N1P2: *F*(1, 96) = 1.167, *p* = 0.283], which indicated that the amplitude of VEPs of V1 cortex in response to visual stimuli with different orientations was identical before HFS. Subsequently, we analyzed the VEP amplitude alterations at different time point (0–180 min) after versus before repeated HFS for test visual stimuli with orientation at SO and NSO, respectively.

Two-way ANOVA analysis showed that the mean N1P1 and P1N2 of V1-cortical VEPs in response to test visual stimuli with SO (90°) after the end of repeated HFS was significantly different from that before HFS (b1 and b2 combined) [N1P1:F(13, 360) = 125.056, *p* < 0.0001; P1N2:*F*(13, 360) = 106.038, *p* < 0.0001], and this effect had no significant interaction with subject of cats [N1P1: *F*(39, 360) = 0.718, *p* = 0.896; N1P2:*F*(39, 360) = 0.620, *p* = 0.964] (Figure 2, SO). Further LSD (least significance difference) *Post hoc* tests indicated that the mean N1P1 amplitude of VEPs in response to visual stimuli with SO recorded at 0, 15, 30, 45, 60, 75, 90, 105, 120, and 135 min after the end of HFS was significantly increased compared with that before (b1 and b2 combined) HFS (*p* < 0.0001, 0.0001, 0.0001, 0.0001, 0.0001, 0.0001, 0.0001, 0.0001, 0.0001, 0.0001), and the increase reached a peak value around 45 min after the end of HFS and then decreased gradually to a level showing no significant difference from that before HFS at 150, 165 and 180 min (*p* = 0.228, 0.948, 0.508) after the end of HFS (Figure 2, SO). The mean P1N2 amplitude of VEPs in response to visual stimuli with SO recorded at 0, 15, 30, 45, 60, 75, 90, 105, 120, 135, and 150 min after the end of HFS was significantly improved compared with that before (b1 and b2 combined) HFS (*p* < 0.0001, 0.0001, 0.0001, 0.0001, 0.0001, 0.0001, 0.0001, 0.0001, 0.0001, 0.0001, 0.0001), and the improvement reached a peak around 30 min after the end of HFS and then reduced gradually to a value exhibiting no significant difference from that before HFS at 165 and 180 min after the end of HFS (*p* = 0.956, 0.606) (Figure 2, SO).

The repeated HFS also had an evident impact on the amplitude of V1-cortical VEPs in response to test visual stimuli with NSO. Two-way ANOVA indicated that the mean N1P1 and P1N2 of VEPs evoked by visual stimuli with NSO before (b1 and b2 combined) and after HFS displayed significant variation [N1P1: *F*(13, 360) = 87.217, *p* < 0.0001; P1N2: *F*(13, 360) = 60.927, *p* < 0.0001], and this effect had no significant interaction with subject [N1P1: *F*(39, 360) = 0.801, *p* = 0.798; N1P2: *F*(39, 360) = 0.825, *p* = 0.763] (Figure 2, NSO). Further LSD Post hoc test.

showed that the mean N1P1 value of VEPs in response to visual stimuli with NSO recorded at 0, 15, 30, 45, 60, 75, 90, and 105 min after the end of HFS was significantly increased compared with that before (b1 and b2 combined) HFS (*p* < 0.0001, 0.0001, 0.0001, 0.0001, 0.0001, 0.0001, 0.0001, 0.0001). The increase reached a peak at about 60 min after the end of HFS and then dropped down gradually to a value showing no difference from that before HFS at 120, 135, 150, 165, and 180 min after the end of HFS (*p* = 0.397, 0.770, 0.181, 0.663, 0.798) (Figure 2, NSO). The mean P1N2 amplitude of VEPs in response to visual stimuli with NSO recorded at 0, 15, 30, 45, 60, 75, 90, 105, and 120 min after the end of HFS was significantly improved compared with that before HFS (*p* < 0.0001, 0.0001, 0.0001, 0.0001, 0.0001, 0.0001, 0.0001, 0.0001, 0.001), which attained a peak value at about 45 min after the end of HFS and then decreased gradually to a level that was not significantly different from that before HFS at 135, 150, 165 and 180 min after the end of HFS (*p* = 0.951, 0.898, 0.514, 0.947) (Figure 2, NSO).

**Figure 3:**
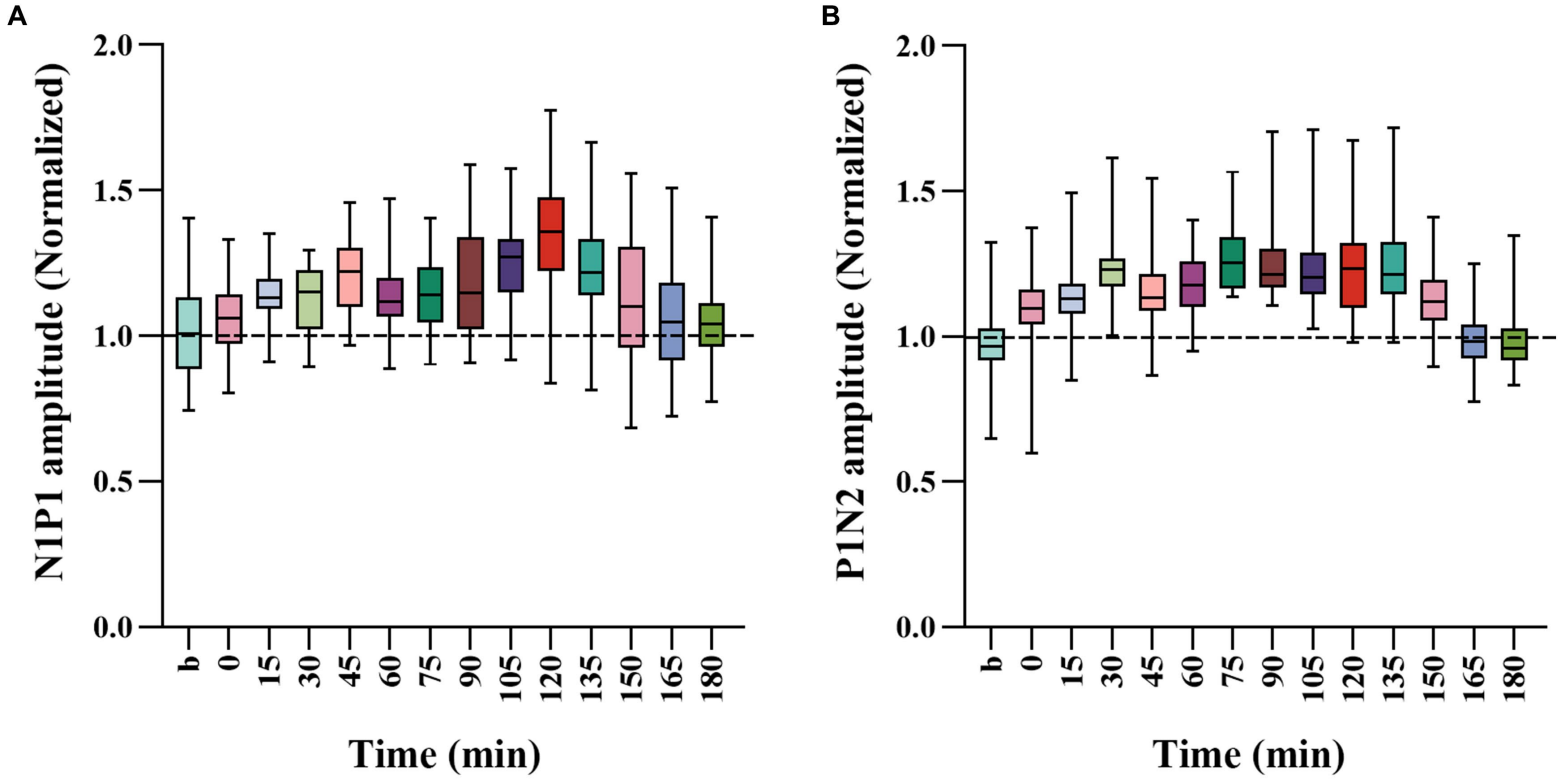
Whisker diagrams showing the mean value across 4 cats for normalized N1P1 **(A)** and P1N2 **(B)** of VEPs in response to visual stimuli with SO (90°) against that with NSO (180°) before and at different time point after the end of HFS. The box plots show the median (middle line within box), 25–75th percentiles (top and lower box edge), minimum and maximum values (whiskers).

To further evaluate the extent of how repeated HFS affected VEPs of V1 cortex in response to visual stimuli with SO and NSO, we, respectively, normalized the amplitude N1P1 and P1N2 of VEPs in response to visual stimuli with SO against that with NSO, and compared the mean normalized N1P1 and P1N2 value across all cats before and after HFS (Figure 3).

One-way ANOVA analysis showed that the normalized N1P1 and P1N2 amplitude of VEPs in response to visual stimuli with SO against that with NSO had significant variation before (b1 and b2 combined) and at different time point after the end of repeated HFS [N1P1: *F*(13, 336) = 7.023, *p* < 0.001; P1N2: *F*(13, 336) = 16.089, *p* < 0.001] (Figures 3A,B). Further LSD *Post hoc* test indicated that the mean normalized N1P1 value measured at 15, 30, 45, 60, 75, 90, 105, 120, 135, and 150 min after the end of HFS was significantly higher than that before (b1 and b2 combined) HFS (*p* < 0.05, 0.036, 0.0001, 0.019, 0.008, 0.01, 0.0001, 0.0001, 0.0001, 0.05) whereas that measured at 0, 165 and 180 min after the end of HFS exhibited no significant variation compared with before HFS (*p* = 0.424, 0.374, 0.501) (Figure 3A). The mean normalized P1N2 value measured at 0, 15, 30, 45, 60, 75, 90, 105, 120, 135, and 150 min after the end of HFS was significantly larger than that before HFS (*p* < 0.05, 0.0001, 0.0001, 0.0001, 0.0001, 0.0001, 0.0001, 0.0001, 0.0001, 0.0001, 0.0001) whereas that measured at 165 and 180 min after the end of HFS had no significant difference from before HFS (*p* = 0.846, 0.874) (Figure 3B).

The comparisons above indicated that repeated HFS induced a long-term potentiation of V1-cortical VEPs in response to visual stimuli with orientation at both SO and NSO, but the effect was stronger, occurred faster and lasted longer for visual stimuli at SO than at NSO.

#### 3.1.2 Effects of repeated LFS on VEPs in V1 cortex

**Figure 4:**
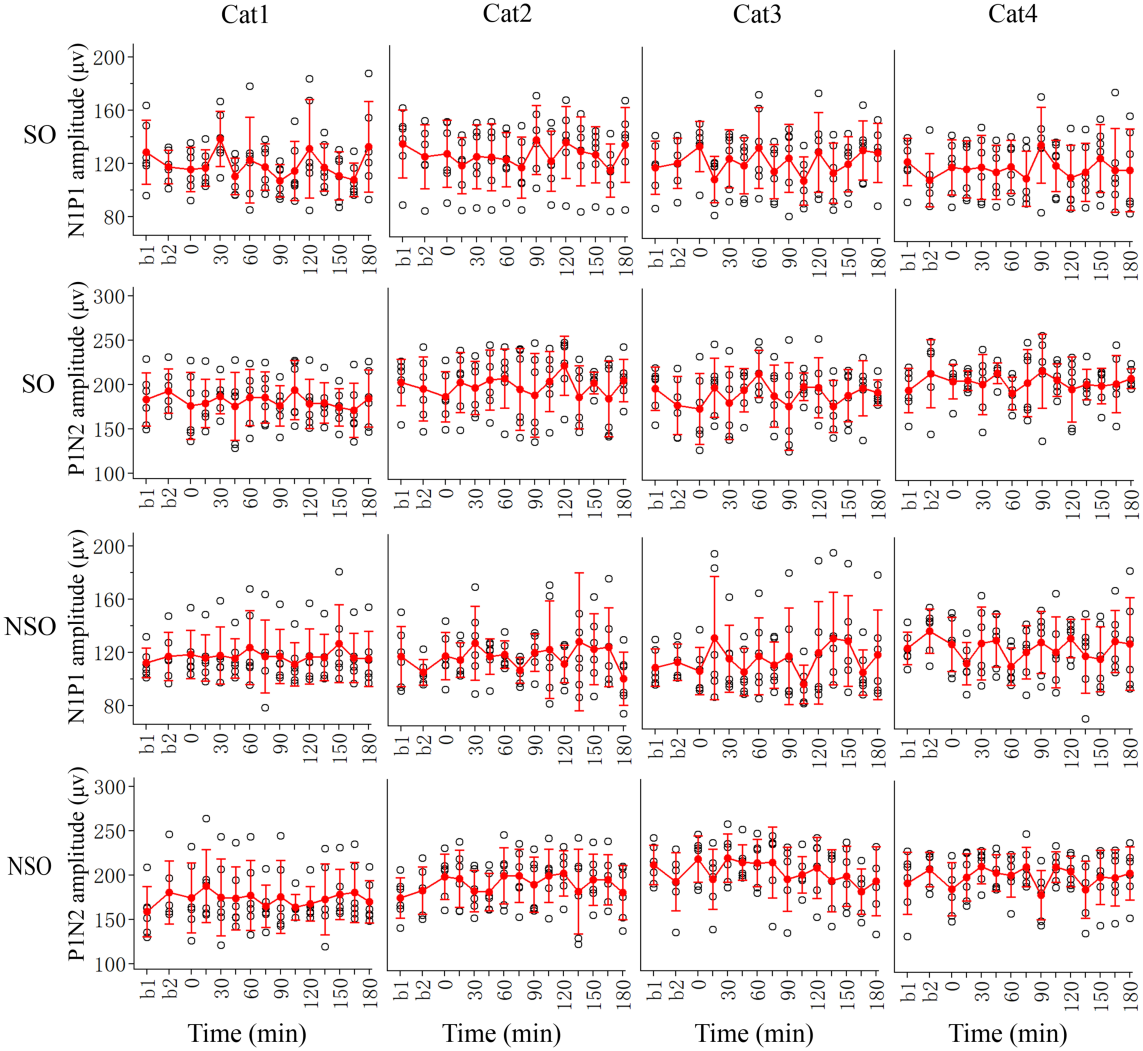
Showing changes in peak-to-peak amplitude N1P1 and P1N2 of VEPs at V1 cortex of 4 cats (Cat1-4) in response to test visual stimuli with stimulated orientation (SO: 90°) and non-stimulated orientation (NSO: 180°) before (b1, b2) and at different time point (0–180 min, with an interval of 15 min) after the end of repeated LFS. The red filled circle with an error bar represents the mean VEP amplitude of N1P1 and P1N2 with standard deviation (SD), and the open black circles represent individual data of N1P1 and P1N2 value from 6 repeated experiments. Each individual data of VEP amplitude is measured across 18 trials (3 iteration with 6 trials per iteration) of test visual stimuli before and at different time point after LFS.

The effect of repeated HFS on the amplitude of VEPs in the V1 cortex could have caused by HFS or simply by repetition of visual stimulation. To examine this possibility, we also observed the effect of repeated low-frequency visual stimulation (LFS) on V1-cortical VEPs in response to test visual stimuli with orientation at SO (90°) and NSO (180°), respectively (Figure 4).

Two-way ANOVA analysis showed that the mean N1P1 and P1N2 amplitude of V1-cortical VEPs in response to visual stimuli with SO recorded before (b1 and b2 combined) and after repeated LFS exhibited no significant variation [N1P1: *F*(13, 360) = 1.017, *p* = 0.435; P1N2:F(13, 360) = 0.647, *p* = 0.813], and this effect had no significant interaction with the subject of cats [N1P1: *F*(39, 360) = 0.632, *p* = 959; P1N2: *F*(42, 360) = 0.443, *p* = 0.999]. Further LSD *Post hoc* test indicated that the mean N1P1 and P1N2 value of VEPs in response to visual stimuli with SO recorded at 0–180 min after the end of LFS had no significant difference from that recorded before (b1 and b2 combined) LFS (N1P1: all *p* > 0.2; P1N2: all *p* > 0.2) (Figure 4, SO).

Similarly, the mean N1P1 and P1N2 value of V1-cortical VEPs in response to visual stimuli with NSO recorded before (b1 and b2 combined) and after repeated LFS exhibited no significant variation either [N1P1: *F*(13, 360) = 0.463, *p* = 0.944; P1N2: *F*(13, 360) = 0.649, *p* = 0.812], and this effect had no interaction with the subject [N1P1: *F*(39, 360) = 0.696, *p* = 0.915; P1N2: *F*(39, 360) = 0.475, *p* = 0.984]. Further LSD *Post hoc* test indicated that the mean N1P1 and P1N2 of VEPs in response to visual stimuli with NSO recorded at 0–180 min after the end of LFS was not significantly different from that recorded before (b1 and b2 combined) LFS (N1P1: all *p* > 0.2; P1N2: all *p* > 0.2) (Figure 4, NSO).

All analysis above indicated that repeated LFS had no significant effect on VEPs in the V1 cortex, and only repeated HFS could induce a LTP-like amplitude increase of VEPs in the V1 cortex although the increase was faster, stronger and lasted longer for VEPs in response to visual stimuli with orientation at SO than at NSO.

### 3.2 Effects of repeated HFS and LFS on the membrane content of AMPA and NMDA receptors in the V1 cortex

Several previous studies show that repeated sensory stimulation can increase the expression of extrasynaptic glutamatergic receptors (Eckert et al., 2013) or induce a neural response potentiation which is blocked by antagonists of NMDA and AMPA receptors (Clapp et al., 2006; Kirk et al., 2010; Clapp et al., 2012). These evidences suggest that HFS-evoked LTP-like potentiation of VEPs in the V1 cortex may involve changes of AMPA and NMDA receptors. Therefore, we assessed the membrane content of the key subunit GluA1 of AMPA receptors and the main subunit NR1 of NMDA receptors in the V1 cortex after repeated HFS or LFS relative to control cats.

**Figure 5:**
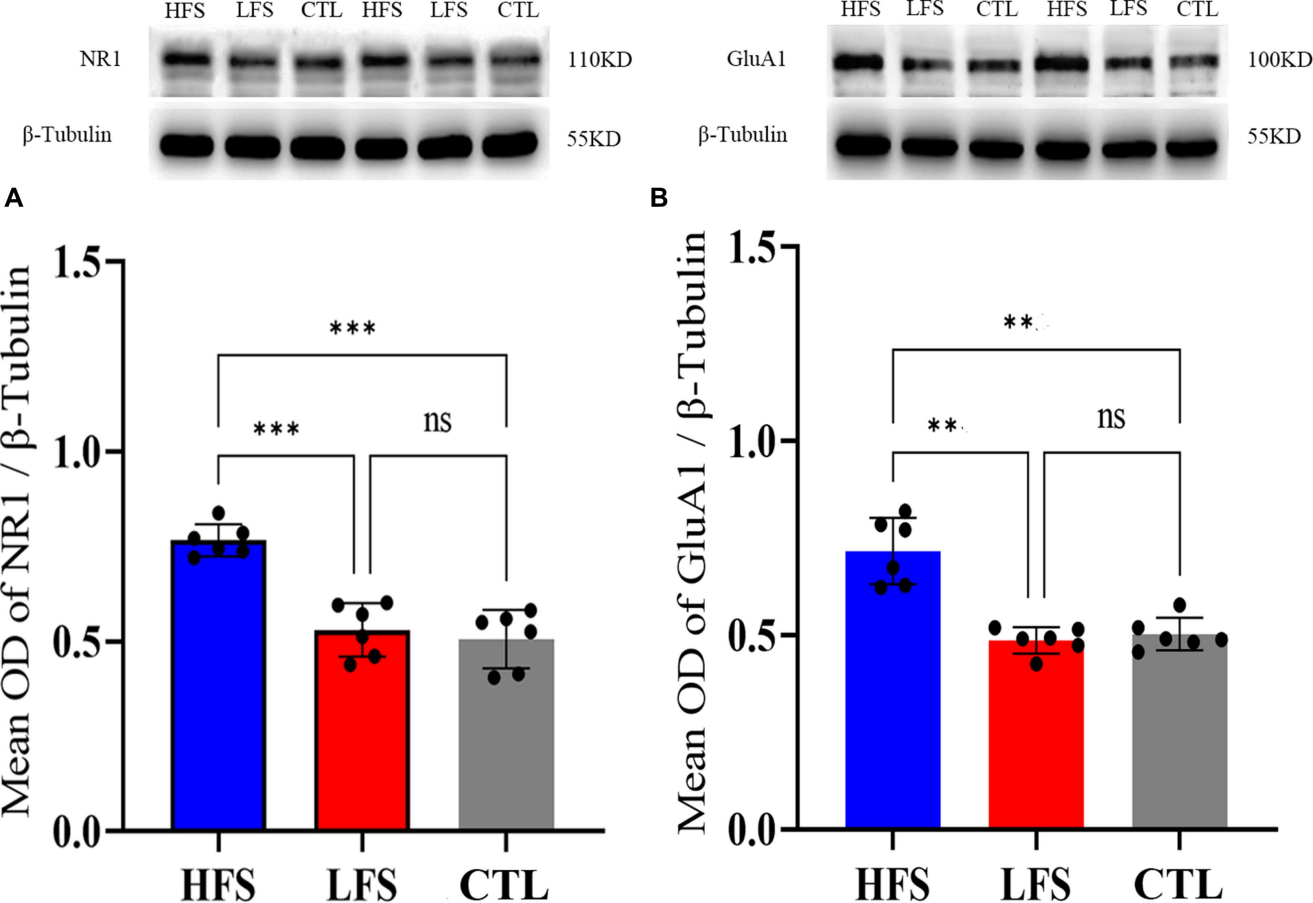
Showing the mean optical density (OD) of Western blot bands of NR1 (the main subunit of NMDA receptors) **(A)** and GluA1 (the key subunit of AMPA receptors) **(B)** normalized against that of β-Tubulin (internal reference) in the V1 cortex after repeated HFS or LFS relative to controls (CTL). The histogram with an error bar represents the mean normalized OD value and SD, and the solid dots on each histogram represent individual data measured from 6 cats. The sample of Western blotting bands of NR1 and GluA1 (upper panel) as well as β-Tubulin (lower panel) are shown on the top of the histogram in **(A,B)**. ***p < 0.0001, **p < 0.001, ns denotes *p* > 0.05.

One-way ANOVA analysis showed that the mean normalized OD value of NR1 against that of β-Tubulin in the V1 cortex exhibited a significant difference among groups after repeated HFS, LFS and controls [*F*(2, 18) = 14.630, *p* < 0.0001]. *Post-hoc* test indicated that the mean normalized OD of NR1 in the V1 cortex after repeated HFS was significantly higher than that after LFS (*p* = 0.0002) and of controls (*p* = 0.0003) whereas the mean normalized OD of NR1 in the V1 cortex after LFS displayed no significant variation from that of controls (*p* = 0.633) (Figure 5A). Similarly, the mean relative OD of GluA1 against β-Tubulin in the V1 cortex exhibited a significant variation among groups after repeated HFS, LFS and controls [*F*(2, 18) = 30.114, *p* < 0.001]. *Post-hoc* test indicated that the mean OD value of GluA1 relative to β-Tubulin in the V1 cortex after HFS was significant larger than that after LFS (*p* < 0.001) and of controls (*p* < 0.001) whereas the relative OD of GluA1 in the V1 cortex after LFS was not significantly different from that of controls (*p* = 0.461) (Figure 5B).

The analysis above indicated that repeated HFS but not LFS could significantly increase the membrane content of glutamatergic AMPA and NMDA receptors in the V1 cortex, which might mediate the LTP-like amplitude increase of VEPs in the V1 cortex after repeated HFS.

## 4 Discussion

### 4.1 The property of LTP-like neural response potentiation induced by visual stimulation

The classic long-term potentiation (LTP) refers to an input-specific and long-lasting increase of postsynaptic potentials evoked by high-frequency electrical stimulation at the presynaptic fibers in *in vitro* studies (Sarvey et al., 1989; Bear et al., 1992; Colbert and Levy, 1993; Kudoh and Shibuki, 1994; Murphy et al., 1997; Volianskis and Jensen, 2003; Miyamoto, 2006). Recent *in vivo* studies report that noninvasive transcranial magnetic stimulation (TMS) (Naro et al., 2015; Chung et al., 2016), direct current stimulation (tDCS) (Ding et al., 2016; Agboada et al., 2020; Zhao et al., 2020; Frase et al., 2021) and even rapidly repeated visual or auditory stimulation (Clapp et al., 2005; Teyler et al., 2005; Ross et al., 2008; Kirk et al., 2010; Lengali et al., 2021) can also induce a LTP-like increase in hemodynamic response or visualy-evoked field potentials (VEPs). However, whether this LTP-like neural response improvement shows a property identical to the classic LTP remains in debate. Some studies show that sensory induced LTP-like improvement of neural response is similar in stimulus-specificity and longevity to the classic LTP (McNair et al., 2006; Ross et al., 2008; Aberg and Herzog, 2012) whereas others report inconsistent plasticity, including an increase in the amplitude of different VEP components, no change or decrease in neural activity as well as lack of stimulus-specific potentiation (Teyler et al., 2005; Lahr et al., 2014; Klöppel et al., 2015; Sumner et al., 2018; Dias et al., 2022; Kleeva et al., 2022). This study examined the effect of repeated HFS versus LFS on VEPs in the V1 cortex, and observed alterations of VEPs in response to test visual stimuli with stimulated orientation (SO) and non-stimulated orientation (NSO) before and at different time (0–180 min) after HFS or LFS. Statistical comparisons show that repeated LFS had no significant effect on VEPs in the V1 cortex, whereas repeated HFS could significantly increase N1P1 and P1N2 amplitude of VEPs in response to visual stimuli with both SO and NSO, but the effect was faster, stronger and lasted longer at SO than at NSO. Our results suggest that repeated HFS but not LFS can induce a long-lasting increase of VEPs in V1 cortex, which is similar to the classic LTP (Bliss and Lomo, 1973; Sarvey et al., 1989; Kirkwood and Bear, 1994; Volianskis and Jensen, 2003) and sensory stimulation-evoked LTP-like response improvement reported in human subjects (Sanders et al., 2018; Valstad et al., 2020; Rygvold et al., 2021, 2022). Nevertheless, our results indicate that HFS-induced VEP-amplitude improvement shows a less specificity to stimulus orientation and can partially generalize to visual stimuli at the other orientations, which differs from LTP-like neural response potentiation observed in human studies (McNair et al., 2006; Ross et al., 2008; Kirk et al., 2010; Clapp et al., 2012; Valstad et al., 2021) and is also unlike the perceptual learning effect with high specificity to trained stimulus parameters (Hua et al., 2010; Lu et al., 2011; Seitz, 2011; Li, 2016; Yan et al., 2018; Yang et al., 2020; Jing et al., 2021; Astorga et al., 2022). Thus, repeated HFS is likely an alternative noninvasive paradigm that can be used to enhance visual cortical excitability and help improve visual ability for patients with impaired vision (Zhang et al., 2014; Yan et al., 2015; Lu et al., 2016).

Reasons leading to the diverse reports about sensory (visual/auditory) induced long-term plasticity of neural activity are poorly understood. At least two factors may contribute. First, different authors have used various stimuli paradigms during LTP induction, such as stimulus type, temporal/spatial frequency and duration (McNair et al., 2006; Ross et al., 2008; Clapp et al., 2012; Eckert et al., 2013; Lahr et al., 2014; Klöppel et al., 2015; Sanders et al., 2018; Rygvold et al., 2021; Dias et al., 2022), which may potentiate the activity of different neuronal populations. Second, methods for measurement of neural response after sensory tetanization varied among different studies (Clapp et al., 2005; Ross et al., 2008; Clapp et al., 2012; Lahr et al., 2014; Valstad et al., 2020; Lengali et al., 2021), which could have measured neural activity at different temporal and spatial scales. Subsequent researches are needed to clarify these possibilities (Dias et al., 2022).

### 4.2 The mechanisms of LTP-like potentiation of VEPs after HFS

The cellular mechanisms underlying the classic LTP induced by *in vitro* electrical stimulation at presynaptic fibers have been extensively investigated over several decades (Bliss et al., 1986; Jay et al., 1995; Murphy et al., 1997; Malenka and Nicoll, 1999; Lynch et al., 2000; Liu et al., 2004; Zhong et al., 2006; Peng et al., 2010). It is known that trains of high-frequency presynaptic stimulation can trigger releasing of more neurotransmitter glutamate, which will open more AMPA receptors at postsynaptic membrane. Subsequent influx of more sodium ions through AMPA receptors cause a large membrane depolarization and lead to the opening of NMDA receptors, and influx of calcium ions through NMDA channels will trigger a series of cascades that cause an increased expression and assembling of AMPA receptors at postsynaptic membrane and thus mediate LTP process (Miyamoto, 2006; Peng et al., 2010).

How rapidly repeated sensory stimulation induce LTP-like increase of neural response in local field potentials or hemodynamic response remains poorly understood. For the long-lasting improvement of neural response at field potential level after repeated sensory stimulation is totally different from the firing rate reduction of single-unit response after sensory stimulus repetition (Peter et al., 2021; Stauch et al., 2021), this LTP-like neural response potentiation could not be mediated by the mechanism of adaptation. Recent studies reports that rapidly presented visual stimulation can enhance the subunit expression of both AMPA and NMDA receptors in the extrasynaptic regions of the membrane although no significant potentiation of VEPs is observed in the primary visual cortex (Eckert et al., 2013), and tDCS can also improve the membrane content of AMPA and NMDA receptors although the total cellular content has no significant change (Zhang et al., 2022). In addition, other studies have found that the LTP-like neural response potentiation is cancelled by blockers of NMDA receptors (Clapp et al., 2006, 2012). These studies suggest that sensory-induced LTP-like neural response improvement could likely be mediated by a mechanism similar to that in classic LTP process (Miyamoto, 2006; Zhong et al., 2006; Peng et al., 2010). However, no study has provided a direct evidence.

The current study comparatively examined the membrane protein content of the key subunit GluA1 of AMPA receptors and the main subunit NR1 of NMDA receptors in the V1 cortex after repeated HFS relative to that after LFS and in controls. The results showed that the membrane protein content of GluA1 and NR1 in V1 cortex was significantly increased after HFS but not LFS when compared with that in controls. This result provide a direct evidence that cellular mechanism underlying visual stimulation-induced LTP-like neural response potentiation is similar to the classic LTP (Miyamoto, 2006; Peng et al., 2010).

In summary, the results in this study indicate that repeated high- but not low-frequency visual stimulation can evoke a LTP-like improvement of VEPs amplitude in the V1 cortex of cats. Similar to the classic LTP (Sarvey et al., 1989; Bliss and Collingridge, 1993; Miyamoto, 2006; Peng et al., 2010), this LTP-like VEPs potentiation is long-lasting and involve membrane trafficking of AMPA and NMDA receptors. Considering that VEPs measure the membrane potentials from a large population of neurons (Lashgari et al., 2012; Haider et al., 2016; Krishna et al., 2021), LTP-like improvement of field potentials evoked by noninvasive sensory stimulation (Clapp et al., 2006, 2012; Eckert et al., 2013), TMS (Aydin-Abidin et al., 2006; Naro et al., 2015), tDCS (Agboada et al., 2020; Frase et al., 2021; Zhang et al., 2022) and perceptual learning (Sale et al., 2011; Lengali et al., 2021) could be a pooled response of postsynaptic potentiation (classic LTP) across neural network. However, the LTP-like VEPs improvement observed in this study shows a less stimulus-input specificity than the classic LTP (Kelso et al., 1986; Kirkwood and Bear, 1994), suggesting other neuronal plasticity, such as changes in intracortical inhibition (Bear et al., 1992; Field et al., 2021), may engage in the effect generalization process. Further studies are needed to elucidate the underlying mechanisms.

## Data availability statement

The original contributions presented in the study are publicly available. This data can be found here: https://data.mendeley.com/datasets/rk5yfb7xc5/1.

## Ethics statement

All experiments in this study were performed strictly in accordance with the National Institutes of Health Guide for the Care and Use of Laboratory Animals, and conformed to the principles and regulations as described in the ARRIVE guidelines. All experiments and animal treatments were approved by the Ethics Committee of Anhui Normal University (approval No: AHNU-ET 2023015).

## Author contributions

SC: Data curation, Formal analysis, Methodology, Visualization, Writing – original draft, Writing – review & editing. HL: Writing – original draft. CC: Writing – original draft. ZY: Writing – original draft. TH: Writing – original draft, Writing – review & editing.
